# Guided internet-administered self-help to reduce symptoms of anxiety and depression among adolescents and young adults diagnosed with cancer during adolescence (U-CARE: YoungCan): a study protocol for a feasibility trial

**DOI:** 10.1136/bmjopen-2016-013906

**Published:** 2017-01-27

**Authors:** Malin Ander, Anna Wikman, Brjánn Ljótsson, Helena Grönqvist, Gustaf Ljungman, Joanne Woodford, Annika Lindahl Norberg, Louise von Essen

**Affiliations:** 1 Clinical Psychology in Healthcare, Department of Women's and Children's Health, Uppsala University, Uppsala, Sweden; 2 Division of Psychology, Department of Clinical Neuroscience, Karolinska Institutet, Stockholm, Sweden; 3 Pediatric Oncology, Department of Women's and Children's Health, Uppsala University, Uppsala, Sweden; 4 Clinical Education Development and Research (CEDAR), Psychology, College of Life and Environmental Sciences, University of Exeter, Exeter, UK

**Keywords:** adolescence, cognitive behavioural therapy, feasibility study, neoplasms, psychological treatment

## Abstract

**Introduction:**

A subgroup of adolescents and young adults diagnosed with cancer during adolescence reports elevated levels of anxiety and depressive symptoms and unmet needs for psychological support. Evidence-based psychological treatments tailored for this population are lacking. This protocol describes a feasibility study of a guided-internet-administered self-help programme (YoungCan) primarily targeting symptoms of anxiety and depression among young persons diagnosed with cancer during adolescence and of the planned study procedures for a future controlled trial.

**Methods/analysis:**

The study is an uncontrolled feasibility trial with a pre-post and 3-month follow-up design. Potential participants aged 15–25 years, diagnosed with cancer during adolescence, will be identified via the Swedish Childhood Cancer Registry. 30 participants will be included. Participants will receive YoungCan, a 12-week therapist-guided, internet-administered self-help programme consisting primarily of cognitive–behavioural therapy organised into individually assigned modules targeting depressive symptoms, worry and anxiety, body dissatisfaction and post-traumatic stress. Interactive peer support and psychoeducative functions are also available. Feasibility outcomes include: recruitment and eligibility criteria; data collection; attrition; resources needed to complete the study and programme; safety procedures; participants' and therapists' adherence to the programme; and participants' acceptability of the programme and study methodology. Additionally, mechanisms of impact will be explored and data regarding symptoms of anxiety, depression, post-traumatic stress, body dissatisfaction, reactions to social interactions, quality of life, axis I diagnoses according to the Mini International Neuropsychiatric Interview and healthcare service use will be collected. Exploratory analyses of changes in targeted outcomes will be conducted.

**Ethics/dissemination:**

This feasibility protocol was approved by the Regional Ethical Review Board in Uppsala, Sweden (ref: 2016/210). Findings will be disseminated to relevant research, clinical, health service and patient communities through publications in peer-reviewed and popular science journals and presentations at scientific and clinical conferences.

**Trial registration number:**

ISRCTN97835363.

**Strengths and limitations of this study**

- This study will examine the feasibility and acceptability of a guided internet-administered self-help programme and of the planned procedures to evaluate it using qualitative and quantitative methods.
- The main challenges related to the programme and study procedures will be identified and findings will be used to refine the programme and design a controlled trial.
- Development of the guided self-help programme has included extensive involvement and consulting of theoretical, clinical and end-user expertise to ensure its scientific and clinical relevance.
- The main study limitations include the uncontrolled design, the relatively short follow-up and the lack of power to examine efficacy.

## Introduction

During the past decade, adolescents diagnosed with cancer have received increased attention due to the specific challenges experienced in managing the short-term and long-term stressors associated with cancer and its treatment while at the same time transitioning from childhood to adulthood.^1^ ^2^ Adolescence and emerging adulthood^3^ are characterised by rapid physical, social and psychological changes and include developmental challenges related to independence, relationships, education and identity. These challenges are often demanding in themselves and can be further complicated by the cancer experience.^1^ Given the transitions experienced during adolescence and emerging adulthood, it has been hypothesised that people diagnosed with cancer during adolescence may have increased vulnerability to symptoms of anxiety, depression and post-traumatic stress. Indeed, some studies have shown that, compared to community controls^4^ ^5^ and people diagnosed at a younger age,^6^ ^7^ people diagnosed with cancer during adolescence report a lower level of health-related quality of life and a higher level of psychological distress, while others have not found such differences^8^ or have even found lower levels of psychological distress compared to population norms^9^ and controls.^10^ However, across the majority of studies, a subgroup of adolescent and young adult survivors of cancer during adolescence report symptoms of anxiety and/or depression at a clinically relevant level. Importantly, longitudinal studies show that the development of cancer-related symptoms is nonlinear, with people reporting symptoms of anxiety and depression years after the completion of treatment.^11^ ^12^

Studies examining the psychological needs experienced by survivors of adolescent cancer are scarce and those few which have been carried out indicate that the existing needs are often unmet.^13^ ^14^ Access to mental health services and social support, including peer-to-peer interaction, are some of the most common unmet needs reported by adolescents and young adults with cancer.^15^ Despite the unique challenges and needs of adolescents and young adults, age-appropriate psychosocial interventions are lacking.^16–18^

Given the reports of elevated levels of psychological distress after completion of treatment for cancer during adolescence^5–7^and the lack of evidence-based psychological interventions,^16–18^ coupled with difficulties in accessing psychological support services,^13–15^ there is a need to increase access to psychological support after adolescent cancer.^4^ ^15^ Evidence is accumulating that internet-based cognitive–behavioural therapy (ICBT) is effective both clinically and in terms of cost for treatment of depression and anxiety symptomatology in adults^19^ and young people^20^ with effects equivalent to face-to-face CBT.^19^ ^21^ ICBT programmes typically include provision of self-help material via the internet^22^ but differ with regard to the type and nature of support provided to engage the patient with the self-help material, ranging from self-administered (no specific support) to therapist-guided (provision of initial support session providing a rationale and regular scheduled support sessions in which progress and process issues are discussed).^23^ ^24^

The important advantages of ICBT are that it can be delivered at a time and place convenient to the individual and reach people in remote locations^25^ ^26^ and thereby increase access to evidence-based treatments. However, most of the existing ICBT programmes are developed for mental health populations, and smaller effect sizes have been shown for people with physical health conditions than for mental health populations.^27^ Taken together, there is a need for psychological interventions addressing the diverse manifestations of illness-related distress and challenges faced by somatic populations.^28^ ^29^ For example, survivors of adolescent cancer struggle with difficulties related to parental overprotectiveness,^30^ loneliness and isolation,^31–33^ social interaction,^34^ school difficulties and lagging behind,^31^ ^33^ worry related to potential infertility,^34^ health anxiety and fear of recurrence,^31^ ^34^ ^35^ changes in body appearance and self-image,^31^ ^36^ ^37^ and existential thoughts related to losses and threat to life.^34^ ^38^ It may be important to address these difficulties when developing psychological interventions for young people diagnosed with cancer during adolescence in order to improve acceptability, engagement and effectiveness.

Although internet-based psychological self-help may suit the needs of young cancer survivors, only two published studies have investigated the feasibility and preliminary clinical efficacy of ICBT for young people diagnosed with cancer during childhood or adolescence.^39–41^ One study investigated the feasibility of an online CBT group intervention to prevent psychosocial problems among adolescents who had completed treatment for cancer diagnosed during childhood and found high levels of satisfaction with the intervention among both participants and the psychologists administering the intervention.^39^ Another study examined the feasibility and preliminary efficacy of an ICBT programme to reduce symptoms of post-traumatic stress and anxiety among adolescent and young adult survivors of cancer during childhood and found the intervention to be acceptable and efficacious with pre-post medium effect sizes on post-traumatic stress and anxiety which were sustained at 3-month follow-up.^40^ ^41^ However, to the best of our knowledge, no study has evaluated guided ICBT specifically developed to reduce symptoms of anxiety and depression among adolescents and young adults diagnosed with cancer during adolescence who have completed cancer treatment.

The primary aim of the planned study is to investigate the feasibility of a guided internet-administered self-help programme (YoungCan) primarily targeting symptoms of anxiety and depression among young people diagnosed with cancer during adolescence, and of the planned study procedures for a future controlled trial to evaluate the programme's clinical efficacy and cost-effectiveness. The feasibility outcomes concern methodological, procedural and clinical uncertainties,^42–44^ and exploratory analyses of changes in targeted outcomes from baseline to postbaseline assessments will be conducted.

## Methods

This protocol is informed by the TREND Statement Checklist^45^ for reporting non-randomised interventions and the SPIRIT guidelines^46^ for reporting clinical trial protocols.

### Design

The study has an uncontrolled, within-group, with a pre-post and 3-month follow-up design with embedded mixed-method process evaluation. All participants will receive a guided internet-administered self-help programme lasting 12 weeks. Potential participants will be identified via the Swedish Childhood Cancer Registry (the National Quality Registry, initiated in 1982).

### Eligibility criteria

Eligible participants will be aged 15–25 years at study start; have been diagnosed with cancer when aged 13–18 years; have been treated at a paediatric oncology unit in Gothenburg, Lund, Linköping or Umeå in Sweden; have completed successful cancer treatment (according to the paediatric oncology unit if the person is 15–17 years at study start or according to self-report if the person is ≥18 years at study start); be able to read and write text in Swedish; have access to email, the internet and a mobile telephone, and report a need for psychological support. Little data are available to guide the selection of screening measures for this population.^47^ ^48^ As such, no cut-off scores on screening measures will be used to determine inclusion into the study; however, the feasibility of screening procedures will be examined to inform the procedures for a future controlled trial.

Potential participants will be excluded if they currently receive psychotherapy or if they display symptoms of severe depression, active suicidality, psychosis, bipolar disorder and/or alcohol and/or substance abuse in immediate need of treatment (based on a clinical assessment combining information from the 9-item Patient Health Questionnaire (PHQ-9)^49^ and the Mini-International Neuropsychiatric Interview (MINI.)^50^). People excluded on this basis will be guided to appropriate healthcare.

### Sample size

Recent reports recommend sample sizes of 50–60 participants^51^ ^52^ to assess feasibility outcomes and estimate sample size for a definitive trial. However, since the target population in Sweden is very small, coupled with experiences from other successful feasibility studies^53–55^ and recommendations,^56^ we aim to include at least 30 participants in the present study.

### Recruitment, setting and procedure

The names and addresses of potential participants will be identified via the Swedish Childhood Cancer Registry. Internet search engines will be used to identify telephone numbers for those aged ≥18 years. For those aged 15–17 years, telephone numbers will be retrieved from the paediatric oncology units. Invitations to participate will be issued in two stages. First, people whose telephone numbers have been identified will be contacted via telephone, in alphabetical order, by study personnel and will receive brief information about the study. Second, if fewer than 30 people have been included when all people with identified telephone numbers have been contacted, written information about the study will be sent to people whose telephone numbers have not been identified or who have not been reached via telephone. Information will be posted on social media via patient organisations, for example, the Swedish Childhood Cancer Foundation and UngCancer (a non-profit organisation supporting adolescents and young adults affected by cancer in Sweden), to raise awareness about the study.

### Informed consent and eligibility interview

People who have been contacted via telephone or post and who are interested in participating will be directed to a secure website where they can register, provide informed consent and answer questions regarding symptoms of depression (PHQ-9^49^) and Generalized Anxiety Disorder 7-item scale (GAD-7^57^). Written information about the study will be sent to caregivers of people aged 15–17 years who have registered on the website.

**Figure 1:**
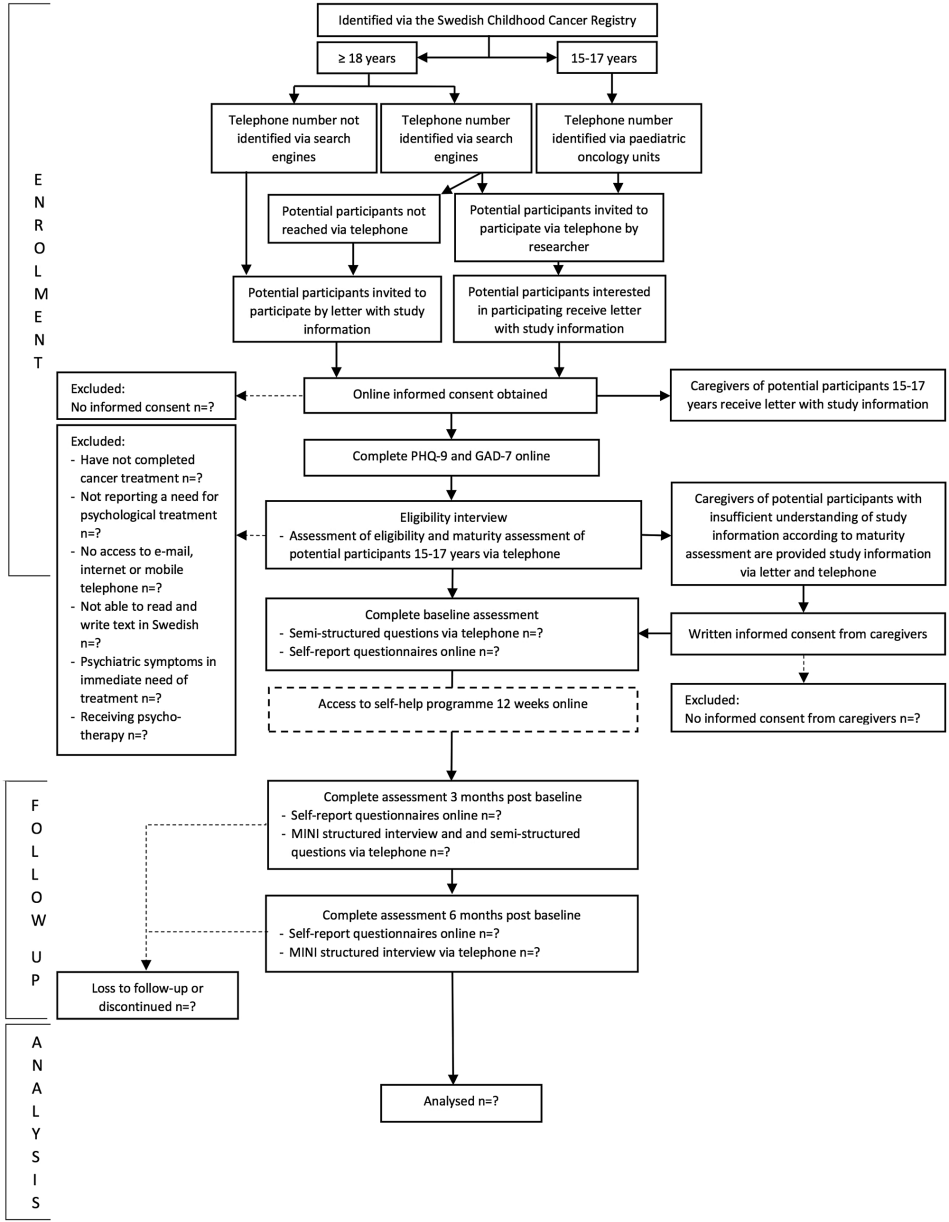
CONSORT diagram. GAD-7, Generalized Anxiety Disorder 7-item scale; MINI, Mini-International Neuropsychiatric Interview; PHQ-9, 9-item Patient Health Questionnaire.

Potential participants will thereafter be contacted via telephone by a clinical psychologist for an eligibility interview to confirm inclusion criteria, that is, completed successful cancer treatment; able to read and write text in Swedish; have access to email, the internet and a mobile telephone; and not currently receiving psychotherapy. The psychologist will also provide a description of the programme, its aims and the types of problems addressed. Potential participants will be asked about their own situation and whether they experience any of the problems addressed in the programme. They will thereafter decide themselves whether they have a need for the support provided, with young adults considered experts of their own perceived needs. Potential participants meeting the inclusion criteria and perceiving a need for psychological support will be interviewed with the MINI^50^ to identify people with psychiatric problems in need of immediate treatment or further examination. People reporting such problems are excluded and guided to appropriate healthcare services. If a potential participant reports suicide ideation but the risk of suicide is assessed as low, the person is offered participation and a safety plan is set up collaboratively. People meeting the eligibility criteria will be offered the opportunity to participate. If the person is aged 15–17 years, an assessment of whether he/she understands what participation implies will be performed to determine whether his/her consent to participate is sufficient or if it needs to be complemented with caregiver consent. If the person appears not to understand the implication of participation, he/she will be asked for permission for study personnel to collect informed consent from his/her caregiver(s). If the caregiver(s) does(do) not consent, participation will not be continued. The flow of participants through the study is shown in figure 1.

### Self-help programme

#### Development

Development of the programme was informed by the UK Medical Research Council (MRC) guidelines for developing and evaluating complex interventions.^44^ Research identifying the specific psychological problems and needs experienced by young people diagnosed with cancer during adolescence^30–38^ and evidence-based cognitive–behavioural models for treating anxiety and depression^58–66^ guided development of the programme. Moreover, development included consultations with paediatric oncology nurses and mental health staff, psychologists with extensive experience of working with CBT with adolescent and young adult mental health populations, psychologists with experience of developing CBT interventions for chronic illness populations, representatives of patient organisations and researchers with experience of developing and evaluating internet-based CBT interventions. Communication agencies were consulted to edit the layout of the material and the secure internet portal—the U-CARE-portal (‘the Portal’)—via which the programme will be delivered. Teachers with extensive experience of educating adolescents reviewed a portion of the text material with regard to readability. Importantly, a group of adolescents and young adults with and without lived experience of cancer during adolescence, formed for the purpose of the present project (n=10), were consulted to review programme content, layout, readability of text material and the procedures planned to test the programme. Additional adolescents and young adults with and without experience of cancer were consulted during development for input on programme content, layout, readability of text material and procedures planned to evaluate the programme.

#### Content

Based on descriptions of self-help^22^ and taxonomies of level of support,^24^ the present programme can be described as guided internet-administered self-help and includes provision of self-help material via the Portal, an initial support session via telephone in which individual problem analyses and idiographic goals are formulated, and regular guidance from a therapist via telephone and the Portal to follow participants' progress and processes.

The programme will be available via the Portal and consists of ICBT, psychoeducation and interactive peer support. The ICBT is considered the core of the programme and is organised into six chapters: introduction, depressive symptoms, anxiety and worry, post-traumatic stress, body dissatisfaction and wrapping up, with the first and last chapters being mandatory. The remaining chapters are each divided into five parts and are assigned to participants based on their needs. Participant needs are determined by individual problem analyses combining baseline questionnaires and interview outcomes, a clinical assessment and a collaborative discussion concerning specific areas and goals the participant would like to prioritise. The clinical assessment follows the Five Areas Assessment Model,^67^ based on the CBT model, providing structure to the assessment.^68^ The chapter(s) best matching participants' problem analyses will be chosen, with additional chapters added later based on participant need and preference. Participants will be encouraged to work with one part of a chapter per week. Each part consists of text material describing treatment components, homework assignments and questionnaires in which homework is reported to the therapist, with text material available as PDF and MP3 files. The majority of the chapters include a PowerPoint presentation, which serves to clarify important treatment principles and help participants make connections between the material and their own lives. Extensive efforts have been made to include ‘common factors’ in the programme and material.^69^ To develop and maintain a therapeutic alliance,^69^ the programme has been developed to engage participants in the text material by including statements of empathy, genuineness and warmth, narratives referring to struggle and recovery, examples to help participants relate the text material to their own lives and personal metaphors for emotional distress.^70^ Online supplementary appendix 1 presents an overview of the ICBT chapters and homework assignments and key references used to develop the ICBT content.

10.1136/bmjopen-2016-013906.supp1 supplementary appendix

#### Guidance from therapists

Participants will work independently with the programme over 12 weeks and receive weekly and at-need guidance from a therapist via messages within the Portal or by telephone. How participants would prefer to be guided is decided collaboratively at study start. Therapists will provide feedback on homework, reinforce progress, validate difficulties and encourage and guide continued work with the ICBT following a standardised treatment manual. Participants will be able to contact their therapist via the Portal or telephone for additional guidance, which will be received within one working day. Given the explorative nature of the study, no maximum time limit for guidance sessions has been set. Participants who do not log in or show low activity in the ICBT will be contacted via text message and/or telephone. During the first week of access to the programme, participants will be contacted via telephone by a therapist for an individually tailored individual problem analysis and formulation of idiographic treatment goals (approximately a 30 min session). On the basis of this analysis, participants will be recommended the ICBT chapters that best match their problems.

#### Optional support functions

Participants will have access to a number of optional support functions built into the ICBT programme, including a library, interaction with other participants and a Questions and Answers (Q&A) function. The library consists of written texts, also available as MP3 files, about common problems and concerns related to cancer during adolescence, alongside video interviews with people with lived experience of cancer during adolescence. Further, the programme provides the possibility to interact with other participants via a chat room, a discussion forum, a private one-to-one messages function and a diary function. Interactions are monitored by study personnel to detect potential risk to self or inappropriate communication, with participants fully informed on study entry and when accessing the chat room for the first time. If risk of suicide/self-harm is detected, standard safety procedures according to the ethical approval will be followed. Participants will also be able to pose questions via a Q&A function, receiving an answer from a study therapist within one working day. Finally, weekly group chat sessions with a study therapist will be offered. Each additional support function is optional and participants do not receive recommendations regarding optimal level of engagement with these additional support functions.

#### Participant adherence

Number of logins, opened ICBT items and completed homework assignments are continuously logged and used to assess participants' adherence to the ICBT. Number of chat sessions, forum posts, private messages, diary posts, questions in the Q&A function and visits to the chat room, forum, diary, library and Q&A functions are logged to examine feasibility and acceptability of optional support functions.

#### Therapist training and adherence

Before study-start, all therapists will take part in a one-day workshop on the programme by the first author (MA), who was part of the programme development team. Therapists will receive weekly group clinical supervision focusing on case discussions, skills development and at-need supervision by a licensed psychologist with experience of working with young people and CBT. Written communication between participants and therapists will be logged and continuously reviewed by the clinical supervisor to assess therapists' adherence to the ICBT protocol and competence in delivering the ICBT. A 15% sample of the communication between therapists and participants within the programme will be reviewed for therapists' adherence to the ICBT protocol and competence in delivering the ICBT according to an adherence measure developed for the YoungCan programme. Findings will be used to identify areas in the programme and therapists training that require modification and/or further development.

### Outcome measurements

#### Feasibility outcomes

The feasibility outcomes concern methodological, procedural and clinical uncertainties^42–44^ related to recruitment and eligibility criteria, data collection, attrition, resources needed to complete the study and programme, safety procedures and process evaluation including participants' and therapists' adherence to the programme, participants' acceptability of the programme and of the study methodology, and exploration of mechanisms of impact. A summary of feasibility outcomes is provided in table 1. In addition, progression criteria, where applicable, which will be used to determine whether revisions should be considered before proceeding to a controlled trial,^43^ are presented.

**Table 1:** Feasibility outcomes and progression criteria

Outcome	Evaluation	Progression criteria to controlled trial*
Recruitment and eligibility	Number of people identified via the Swedish Childhood Cancer Registry, invited via telephone, invited via letter, assessed for eligibility	No criteria set
	Percentages of people interested in participation, assessed for eligibility, meeting the inclusion criteria and included, of the total number identified	≥15% interested in participating of the total number identified≥10% included of the total number identified
	Ambiguities regarding eligibility criteria	No criteria set
	Numbers scoring above/under recommended cut-offs on clinical outcome measures	No criteria set
	Reasons for ineligibility	No criteria set
	Reasons for non-participation	No criteria set
Data collection	Percentage of participants completing assessments	70% answering all questions at all assessments
	Numbers of missing items relating to clinical, psychological and health economics outcomes	No criteria set
	Types and number of potential uncertainties in diagnostic interviews	No criteria set
Attrition	Rates of dropout from study and programme	No criteria set
Resources needed to complete the study and the programme	Length of time for: Participants to work through the programme Participants to complete questionnaires and interviews Therapists to deliver the programme Study personnel to administer the study	No criteria set
Safety procedures	Ambiguities regarding standard safety procedures Types and numbers of measures undertaken to assure patient safety Types and numbers of unforeseen safety issues	No criteria set
Participants’ adherence to the ICBT and use of optional support functions	Number of: Logins Opened ICBT items, completed homework assignments Chat sessions, forum posts, private messages, diary posts, questions in the Q&A function Visits to the chat room, forum, library and Q&A functions	70% completing the introduction ICBT chapter, and the first two parts of an individually assigned chapter
Therapists’ adherence to programme	Content of online written therapist-participant communication	No criteria set
Participants’ acceptability of programme and data collection and exploration of mechanisms of impact	Reasons for poor attendance and withdrawal from study and programme	No criteria set
	Impressions and experiences of working with the programme (including positive and negative consequences) and of completing questionnaires and interviews	70% of participants using the programme reporting that it is helpful <1 participant reporting substantial negative consequences related to participation in the study and/or programme

*If one or more criteria are not met, revisions should be considered before proceeding to a controlled trial.

ICBT, internet-based cognitive–behavioural therapy; Q&A, Question and Answer function.

#### Psychological and health economics outcomes

The feasibility of using a number of psychological and health economics measurements will be examined to assess whether or not to include them in a controlled study. The PHQ-9^49^ will be used to measure symptoms of depression and the GAD-7^57^ to measure anxiety symptoms. Body dissatisfaction will be measured with the Body Image Scale (BIS)^71^ and post-traumatic stress symptoms using the Posttraumatic Stress Disorder Checklist—Civilian version (PCL-C).^72^ Reactions to social interaction situations will be measured with the Social Interaction Anxiety Scale (SIAS).^73^ The EuroQol EQ-5D^74^ will be used to calculate quality-adjusted life years. Aspects regarding use of healthcare services, employment, absence and sick leave will be examined with a modified short version of the Trimbos and Institute of Medical Technology Assessment Cost Questionnaire for Psychiatry (TiC-P),^75^ assessing direct and indirect medical costs and indirect non-medical costs.

#### Demographics and clinical variables

Data on age, gender, diagnosis and date of first diagnosis will be collected via the Swedish Childhood Cancer Registry. Self-reported information on education, employment status (including studying), ethnicity, relationship status, family situation, housing situation, cancer recurrence including date, date of end of treatment, type of treatment, presence and type of late effects and potential receipt of psychological treatment before and after diagnosis will be collected at baseline.

### Data collection

Psychological and health economics-related self-reported data will be collected at the eligibility interview, the baseline assessment and the assessments 3 and 6 months after the baseline assessment. Data will be collected either via telephone or online via the Portal. Prompts will be sent via text message and reminders will be sent via text message or provided by telephone. The outcome measures and mode of administration, at each assessment, are shown in table 2.

**Table 2:** Outcome measures at each assessment

Measure	Eligibility interview	Baseline assessment	Assessment at 3 months postbaseline	Assessment at 6 months postbaseline	Mode of administration
Demographics and clinical variables		x			Online
PHQ-9	x		x	x	Online
GAD-7	x		x	x	Online
BIS		x	x	x	Online
PCL-C		x	x	x	Online
SIAS		x	x	x	Online
EQ-5D		x	x	x	Online
Modified TiC-P		x	x	x	Online
MINI		x	x	x	Telephone

BIS, Body Image Scale; EQ-5D, EuroQol 5-dimension questionnaire; GAD-7, Generalized Anxiety Disorder 7-item scale; MINI, Mini-International Neuropsychiatric Interview; PHQ-9, 9-item Patient Health Questionnaire; PCL-C, Posttraumatic Stress Disorder Checklist-Civilian version; SIAS, Social Interaction Anxiety Scale; TiC-P, Trimbos and Institute of Medical Technology Assessment Cost Questionnaire for Psychiatry.

Data collection via telephone at 3 and 6 months post baseline will be performed by psychologists and clinical psychology students not otherwise involved in the study. Potential participants/participants who report psychiatric symptoms in need of treatment or further psychiatric examination will be guided to appropriate healthcare and if the person is aged 15–17 years, their caregiver(s) will be informed.

### Process evaluation

To assess the programme's relevance in terms of participants' acceptability of and adherence to the programme, and to generate hypotheses regarding mechanisms of impact, semistructured interviews will be conducted via telephone at baseline and 3 months postbaseline. At baseline, participants will be asked to describe problems, hypotheses about problem development and maintaining factors, experiences of psychological treatment and expectations of the programme. Three months after baseline, participants will be interviewed regarding impressions and experiences of working with the programme and of completing questionnaires and interviews. Moreover, participants' activity in the Portal will be logged continuously to assess adherence to the ICBT and the feasibility and acceptability of optional support functions. Non-attendees, that is, those included but who do not attend the first week telephone session or complete the ICBT introduction chapter and poor attendees, that is, those included who attend the first week telephone assessment and complete the ICBT introduction chapter but fail to complete more than the first part of the assigned ICBT chapter, will be asked about their reasons for not adhering to the programme and what a programme should include. To explore non-acceptability of the programme and methods, participants who choose to exit the study will be asked if they agree to be interviewed about their reasons for exiting and what a programme should include. Interviews will be conducted via telephone by clinical psychology students not involved in delivering the programme and are anticipated to last ∼45 min. If the participant permits, the interview will be digitally recorded. Participants will be given the option to complete the interview over one or two telephone sessions.

### Data analyses

Data analyses will primarily be descriptive and will address the primary outcomes relating to the feasibility of the programme and study methods.

#### Quantitative analyses

The CONSORT diagram will be used to illustrate participant flow. Numbers of potential participants identified via the Swedish Childhood Cancer Registry, invited via telephone, invited via letter, assessed for eligibility, eligible and included will be reported. The percentages of potential participants willing to undergo eligibility assessment of the total number invited, of potential participants overall meeting the eligibility criteria out of the total number invited and of participants overall enrolled in the study out of the total number invited will be calculated with exact 95% CIs. Reasons for ineligibility, ambiguities regarding eligibility criteria, reasons for non-participation and numbers included via telephone versus postal letter will be reported. To generate hypotheses regarding appropriate screening measures, numbers scoring above/under cut-offs recommended for similar populations on clinical outcome measures related to problems addressed in the ICBT will be reported.

Follow-up rates and numbers of missing items relating to clinical outcomes will be calculated with 95% CIs alongside means and SDs for the number of reminders via text message and telephone. Potential assessment uncertainties in diagnostic interviews will be reported alongside means and SDs for time taken to complete questionnaires and interviews. Descriptive statistics including the means and SDs or medians and IQRs and change scores for each outcome measurement at the eligibility interview, baseline and 3 and 6 months postbaseline will be reported. Attrition proportions (both programme and study dropout) will be reported with 95% CIs.

Means, SDs and frequencies for each Portal activity relating to adherence to the ICBT including logins; opened ICBT items and completed homework assignments will be reported; alongside means, SDs and frequencies for each Portal activity relating to the use of optional support functions including chat sessions; forum posts; private messages; diary posts; questions in the Q&A function; visits to the chat room, forum, diary and Q&A functions, and visits to the library will be reported. Means, SDs and frequencies of participant–therapist contact within the Portal and via telephone will be reported and therapist adherence measures will be summarised with means and SDs and collated in total and by therapist.

Means and SDs for the length of time taken for participants to work through the programme; for participants to complete the eligibility assessment, baseline, follow-up assessments and interviews; for therapists to deliver the programme; for therapist training and supervision, and for project personnel to administer the data collection procedures from the invitation to the 3-month follow-up, will be reported to estimate the required resources and to assess the feasibility of the programme and suggested methods. Finally, potential ambiguities regarding standard safety procedures, types and numbers of measures undertaken to assure patient safety and types and numbers of unforeseen safety issues will be reported.

#### Qualitative analyses

Answers to semistructured questions will be recorded, transcribed verbatim, checked for accuracy by the first author (MA) and thereafter analysed with manifest content analysis.^76^ The same analysis will be used to analyse the communication in the interactive functions of the programme. Two researchers will be involved in all stages of the analysis to enable reflection and discussion during the entire process.^76^ Tentative categories and subcategories will be presented to an additional researcher and people with lived experience of cancer during adolescence to discuss the credibility of how data have been sorted. The necessary revisions will be made and thereafter categories and subcategories will be presented to study participants to explore whether the categorisation and labelling of data accurately illustrates participants' experiences.

## Ethics and dissemination

The study will be conducted in accordance with the Helsinki Declaration to ensure the welfare and rights of participants. The participants' confidentiality will be guaranteed and consideration will be given to their integrity, dignity and vulnerability. Informed consent will be collected to ensure that participants are aware of the conditions of participation. Participants exiting the study will be reminded about their rights to exit without giving any reasons. All data will be handled in accordance with the Swedish Personal Data Act (1998:204) and Patient Data Act (2008:355). Potential participants will be assigned a user ID to de-identify data. Data not collected via the Portal will be stored in a locked filing cabinet, only accessible to the researchers, with participant personal information stored separately from de-identified data. All data collected via the Portal will be stored on secure servers at Uppsala University. Personal data and user generated data are stored in different databases on different servers. The Portal secures de-identification of data and prevents unauthorised persons from connecting data from different Portal databases.

Potential adverse events and negative effects of the programme and study will be explored and reported. Assessments of whether participants present symptoms requiring psychological services that cannot be offered within the scope of this study will be made at three assessments. Moreover, online written communication will be monitored to detect participants who signal a need for more extensive services, including risk of harm to self, and with the management of potentially harmful or destructive online communication. Standard safety procedures for managing such instances have been developed, with participants fully informed of these procedures on study entry. People with a need for psychological services that cannot be met within this study will be guided to appropriate healthcare services.

## Discussion

Evidence-based interventions to reduce psychological distress following cancer during adolescence are lacking and the psychological needs of survivors of adolescent cancer are often unmet.^13^ ^14^ Adolescents and young adults diagnosed with cancer during adolescence are a relatively small and geographically dispersed group, and ICBT may have the potential to overcome many of the barriers to psychological support that have been reported by this population.^77^ However, evidence indicates that to be effective, ICBT programmes may need to address the unique experiences of people with physical health conditions and comorbid anxiety and depression.^28^ This study will test the feasibility of YoungCan, an internet-based self-help programme developed to reduce anxiety and depressive symptoms among young people diagnosed with cancer during adolescence. Testing the feasibility of complex interventions and trial methods is strongly recommended to estimate important parameters and answer key uncertainties required to design controlled trials.^40^ ^41^ Given the novelty of the programme and the few intervention studies conducted with the target population, assessing the acceptability and feasibility of the programme and methods used is of great importance in guiding programme refinements and informing the planning of an efficacy trial, including the generation of hypotheses regarding mechanisms of impact.

If the programme proves feasible, our aim is to evaluate it in a controlled trial, and if it proves effective we will aim to implement the programme within routine follow-up care of young persons treated for cancer during adolescence. Guided internet-administered self-help tailored to young people diagnosed with cancer during adolescence has the potential to decrease suffering and costs for the individual as well as costs for society as a whole.
