# Supplementary material for: Guided internet-administered self-help to reduce symptoms of anxiety and depression among adolescents and young adults diagnosed with cancer during adolescence (U-CARE: YoungCan): a study protocol for a feasibility trial
Source: BMJ Open. 2017 Jan 27;7(1):e013906. doi: 10.1136/bmjopen-2016-013906 (PMC5278290; doi:10.1136/bmjopen-2016-013906)
Supplement: supplementary appendix [file bmjopen-2016-013906supp_appendix.pdf]

Supplementary appendix 1: Overview of the ICBT content

ICBT chapter	ICBT content: *Text/audio/PowerPoint material*	ICBT content: *Homework*	Key references
INTRODUCTION (mandatory)			
	• Information about the practicalities of the ICBT component and the general principles of CBT<br>• Education about psychological distress following cancer during adolescence<br>• Brief description of the content in each chapter	➢ Answer questions to assess understanding of CBT principles and to explore doubts and concerns about working with the ICBT component<br>➢ Describe own cancer-related distress	[1, 2]
SYMPTOMS OF DEPRESSION (optional)			[3-6]
PART 1	*Depression and the vicious cycle*		
	• Information about depressive symptoms following cancer<br>• Description of the behavioural activation model to improve mood and the regulating mood through activity approach<br>• Explanation of the activity log and positively and negatively reinforced behaviours	➢ Answer questions to explore potential doubts and concerns about the chapter content<br>➢ Identify life circumstances and secondary problem behaviours that may impact on mood<br>➢ Set specific and concrete goals<br>➢ Log activities and mood<br>➢ Explore activities that improve function and/or mood	
PART 2	*Discover patterns in your behaviour and do more of things you like*		
	• Description of patterns of avoidance and escape behaviours and their consequences<br>• Education about the importance of feeling closeness<br>• Explanation of focused activation	➢ Identify idiographic patterns of avoidance and escape<br>➢ Look for procrastination behaviours and feelings of closeness<br>➢ Schedule activities and assess their outcomes	

ICBT chapter	ICBT content<br>*Text/audio/PowerPoint material*	*Homework*	Key references
PART 3	*Challenge avoidance*		
	• Explanation of avoidance modification and strategies to facilitate goal-directed behaviour<br>• Information about the association between depressive feelings and depressive thoughts<br>• Description of rumination as a common escape and avoidance behaviour and of a mindfulness-based approach to thoughts	➢ Identify avoidance behaviours<br>➢ Prioritise and make activation plans to break avoidance patterns<br>➢ Observe and analyse the context of thoughts<br>➢ "I have a thought" cognitive defusion exercise<br>➢ Monitor and analyse consequences of rumination	
PART 4	*Get out of your head*		
	• Information about the importance of continuing with focused activation<br>• Explanation of mindfulness training as a way to challenge rumination and to engage in life<br>• Description of values as a way to guide life decisions	➢ Challenge rumination with attention to experience – mindfulness training<br>➢ Explore values<br>➢ Schedule activities and assess their outcomes	
PART 5	*Review and plan ahead*		
	• Education about the role of continued practice of learned skills<br>• Normalisation of setbacks and description of strategies to prevent relapses into depressive behaviours	➢ Review treatment goals, summarise main lessons learnt, identify strengths and challenges<br>➢ Plan for maintaining gains and working towards goals, handling setbacks and preventing relapses	
WORRY AND ANXIETY(optional)			[5-13]
PART 1	*Notice when you worry*		
	• Information about worry and fear following cancer<br>• Education about the functions of worry, fear and anxiety, and normalisation of typical experiences<br>• Brief description of worry awareness training and a short version of applied relaxation	➢ Answer questions to explore doubts and concerns about the chapter content<br>➢ Set specific and concrete goals<br>➢ Train worry awareness<br>➢ Practice applied relaxation	

ICBT chapter	ICBT content<br>*Text/audio/PowerPoint material*	*Homework*	Key references
PART 2	*View your thoughts as thoughts*		
	• Education about the function of thoughts and different types of worrying thoughts<br>• Description of cognitive defusion and mindfulness training as alternatives to worry and rumination	➢ Answer questions to explore doubts and concerns about the chapter content<br>➢ Try a cognitive defusion exercise<br>➢ Observe and label worrying thoughts in daily life<br>➢ Continue with relaxation exercises	
PART 3	*Get out of your head*		
	• Explanation of worry exposure, stimulus control and problem solving<br>• Description of how to determine whether to use problem solving or worry exposure	➢ Experiment with worry time, worry exposure and problem solving<br>➢ Continue with relaxation exercises	
PART 4	*Worry behaviours*		
	• Education about different types of worry behaviours and explanation of how to challenge them<br>• Description of mindfulness-based observation of thoughts, emotions and physical sensations to aid worry exposure	➢ Map and challenge worry behaviours<br>➢ Continue experimenting with worry time, worry exposure and problem solving<br>➢ Continue with relaxation exercises	
PART 5	*Review and plan ahead*		
	• Information about the role of continued practice of learned skills<br>• Normalisation of setbacks and description of strategies to prevent relapses into maladaptive worry behaviours	➢ Review treatment goals, summarise main lessons learnt and identify strengths and challenges with regard to continuing work independently<br>➢ Plan for maintaining gains and working towards goals, handling setbacks, and preventing relapses	
DISSATISFACTION WITH BODY AND APPEARANCE (optional)			[5, 14-19]
PART 1	*Understanding your dissatisfaction*		
	• Information about dissatisfaction with appearance following cancer and normalisation of typical experiences	➢ Answer questions to explore doubts and concerns about the chapter content<br>➢ Describe own bodily changes	

ICBT chapter	ICBT content<br>*Text/audio/PowerPoint material*	*Homework*	Key references
	• Explanation of the role of attention and of avoidance and safety behaviours in maintaining dissatisfaction and fears<br>• Explanation of the rationale for systematic exposure to fear-eliciting stimuli	➢ Describe thoughts, feelings, and avoidance and safety behaviours in situations affected by dissatisfaction	
PART 2	*View your thoughts as thoughts – not facts*		
	• Description of a mindfulness-based approach to thoughts<br>• Explanation of self-focus and its consequences and the rationale for focusing attention externally and on the present moment	➢ Answer questions to explore doubts and concerns about the ICBT and homework<br>➢ Monitor thoughts<br>➢ Try a cognitive defusion exercise<br>➢ Practice focus exercises	
PART 3	*Challenge your dissatisfaction*		
	• Information about the functional aspects of anxiety and avoidance<br>• Explanation of graded systematic exposure to fear eliciting situations or events with response prevention as a means to living a valued life	➢ Create an exposure hierarchy<br>➢ Conduct systematic graded exposure exercises<br>➢ Assess and reflect on outcomes from exposure exercises	
PART 4	*Continue to challenge your dissatisfaction*		
	• Explanation of the role of continued exposure to feared stimuli and of reflection on main lessons learnt and potential need for hierarchy revision	➢ Continue with exposure exercises<br>➢ Assess and reflect on outcomes and main lessons learnt from exposure exercises	
PART 5	*Review and plan ahead*		
	• Information about the role of continued practice of learned skills<br>• Normalisation of setbacks and description of strategies to prevent relapses into maladaptive avoidance	➢ Review treatment goals, summarise main lessons learnt and identify strengths and challenges with regard to working independently<br>➢ Plan for maintaining gains and continue work towards goals, handling setbacks and preventing relapses	

ICBT chapter	ICBT content<br>*Text/audio/PowerPoint material*	*Homework*	Key references
TRAUMATIC STRESS SYMPTOMS (optional)			[5, 6, 20-22]
PART 1 *Crisis reactions*	• Information about cancer as a potentially traumatic event and description of common psychological reactions to normalise typical experiences<br>• Explanation of the rationale for gradual exposure to cancer-related internal and external stimuli	➢ Answer questions to explore doubts and concerns about the chapter content<br>➢ Set specific and concrete goals<br>➢ Identify and describe cancer-related distress	
PART 2 *Looking back*	• Description of a mindfulness-based approach to thoughts and emotions<br>• Explanation of consequences of avoidance of internal processes<br>• Explanation of the role of looking back and sorting cancer-related feelings and thoughts	➢ Explore thoughts and feelings related to cancer through a detailed writing assignment. Participants are asked to write in present tense, first person and describe experiences in detail<br>➢ Monitor reactions to cancer-related reminders	
PART 3 *The traces in you*	• Information about common difficulties related to returning to normal life after the end of cancer treatment and to changes in self-image<br>• Explanation of the rationale for increasing activities that can improve mood and increase self-knowledge	➢ Reflect on the impact of cancer on self-image and life through a writing assignment<br>➢ Identify personal strengths through a writing assignment<br>➢ Monitor reactions to cancer-related reminders and conduct graded exposure exercises relating to reminders<br>➢ Explore activities that can help improve function and mood and learn more about oneself	
PART 4 *Looking ahead*	• Information about worry and anxiety related to the future and positive consequences of finding courage to plan for a future	➢ Monitor reactions to cancer-related reminders and graded exposures to reminders<br>➢ Explore future-related anxiety and hopes for the future through a writing assignment	

**ICBT chapter**	**ICBT content** *Text/audio/PowerPoint material*	***Homework***	**Key references**
	• Explanation of how values can be used as a direction and guide for decisions	➢ Explore own values<br>➢ Experiment with activities that can help improve function and mood	
PART 5	*Review and plan ahead*		
	• Information about the role of continued practice of learned skills<br>• Normalisation of setbacks and description of strategies to prevent relapses into maladaptive behaviours	➢ Review treatment goals, summarise main lessons learnt and identify strengths and challenges with regard to continuing to work independently<br>➢ Plan for maintaining gains and continue work towards goals, handling setbacks and preventing relapses	
WRAPPING UP (mandatory)			[1]
	• Explanation of the role of summarising treatment and acknowledging own efforts<br>• Description of strategies to maintain gains and continue to make progress<br>• Normalisation of setbacks and description of how they can be handled	➢ Review own progress and main lessons learnt<br>➢ Plan for continued progress and maintaining gains<br>➢ Make an action plan to handle setbacks and prevent relapses into problem behaviours	

References

1. Farmer RF, Chapman AL. *Behavioral interventions in cognitive behavior therapy: practical guidance for putting theory into action*. Washington, DC: American Psychological Association, 2008.
2. Lukens EP, McFarlane WR. Psychoeducation as evidence-based practice: considerations for practice, research and policy. *Brief Treatment & Crisis Intervention* 2004;4(3):205–25.
3. Martell CR, Dimidjian S, Herman-Dunn R, *et al*. *Behavioral activation for depression: a clinician's guide*. New York: Guilford Press, 2010.
4. Addis M, Martell CR. *Overcoming depression one step at a time: the new behavioral activation approach to getting your life back*. Oakland, CA: New Harbinger Publications, 2004.
5. Wells A. *Metacognitive therapy for anxiety and depression*. New York: Guilford Press, 2008.
6. Hayes S, Strosahl, K, Wilson K. *Acceptance and commitment therapy: an experiential approach to behavior change*. New York: Guilford Press, 1999.
7. Borkovec TD, Alcaine OM, Behar E. Avoidance theory of worry and generalized anxiety disorder. In: Heimberg RG, Turk CL, Mennin DS, eds. *Generalized anxiety disorder: advances in research and practice*. New York: Guilford Press, 2004:77–108.
8. Robichaud M. Generalized anxiety disorder: targeting intolerance of uncertainty. In: Simos G, Hofmann, SG, eds. *CBT for anxiety disorders: a practitioner book*. Hoboken: Wiley-Blackwell, 2013:57–86.
9. Fardell JE, Thewes B, Turner J, *et al*. Fear of cancer recurrence: a theoretical review and novel cognitive processing formulation. *J Cancer Surviv* 2016;10(4):663–73.
10. Thewes B, Brebach R, Dzidowska M, *et al*. Current approaches to managing fear of cancer recurrence; a descriptive survey of psychosocial and clinical health professionals. *Psychooncology* 2014;23(4):390–6.
11. Taylor S, Asmundson JG. *Treating health anxiety: a cognitive–behavioral approach*. New York: Guilford Press, 2004.
12. McCracken LM. "Attention" to pain in persons with chronic pain: a behavioral approach. *Behav Ther* 1997;28(2):271–84.
13. Wuthrich VM, Rapee RM, Cunningham MJ, *et al*. A randomized controlled trial of the Cool Teens CD-ROM computerized program for adolescent anxiety. *J Am Acad Child Adolesc Psychiatry* 2012;51(3):261–70.
14. Wong J, Gordon EA, Heimberg RG. Cognitive-behavioral models of social anxiety disorder. In: Weeks JW, ed. *The Wiley Blackwell handbook of social anxiety disorder*. Chichester, UK: John Wiley & Sons, Ltd, 2014:3–23.
15. Gordon D, Wong J, Heimberg RG. Cognitive-Behavioral therapy for social anxiety disorder: the state of the science. In: Weeks JW, ed. *The Wiley Blackwell Handbook of Social Anxiety Disorder*. Chichester, UK: John Wiley & Sons, Ltd, 2014:477–97.
16. Kent G. Understanding the experiences of people with disfigurements: an integration of four models of social and psychological functioning. *Psychol Health Med* 2010;5(2):117–29.
17. Moscovitch, DA. What is the core fear in social phobia? A new model to facilitate individualized care conceptualization and treatment. *Cogn Behav Pract* 2009;16(2):123–34.
18. Pearson A, Heffner M, Follette V, *et al*. *Acceptance and commitment therapy for body image dissatisfaction: a practitioner's guide to using mindfulness, acceptance, and values-based behavior change strategies*. Oakland, CA: New Harbinger Publications, 2010.
19. Veale D, Anson M, Miles S, *et al.* Efficacy of cognitive behavior therapy versus anxiety management for body dysmorphic disorder: a randomised controlled trial. *Psychother Psychosom* 2014;83(6):341–53.
20. Lange A, van de Ven JP, Schriecken B. Interapy: treatment of post-traumatic stress via the internet. *Cogn Behav Ther* 2003;32(3):110–24.
21. Knaevelsrud C, Maercker A. Long-term effects of an internet-based treatment for posttraumatic stress. *Cogn Behav Ther* 2010;39(1):72–7.
22. Cohen JA, Mannarino AP, Deblinger E. *Treating trauma and traumatic grief in children and adolescents*. New York: Guilford Press, 2006.
